# A review of epoxy vitrimer-based thermally conductive composites

**DOI:** 10.1039/d5ra05874k

**Published:** 2025-10-10

**Authors:** Huimei Yao, Xuan Xu, Zipeng Chen, Kai Li, Tao Wang, Shenghua Pei, Jiaojiao Fu, Jiaxin Wang

**Affiliations:** a School of Mechanical and Electronic Engineering, Ji'an College Ji'an 343000 Jiangxi Province PR China 13860202695@126.com

## Abstract

Driven by advancements in integrated and high-power electronic devices, polymer composites featuring high thermal conductivity (TC), recyclability, and dynamic functionalization have emerged as a focal point in thermal management research. Epoxy vitrimer (EV), owing to its covalent adaptable network (CAN), which enables self-healing, recyclability, and network reconstruction, presents a novel paradigm for designing high-performance thermally conductive composites (TCCs). This review systematically reviews the synthesis strategies, exchange mechanisms, and properties of epoxy vitrimer-based thermally conductive composites (EVTCCs) across diverse CAN architectures (*e.g.*, ester, disulfide, imine, *etc.*), while highlighting key advancements and future directions in this evolving field.

## Abstract 2

Pioneering review connecting covalent adaptable networks to high-performance recyclable epoxy vitrimer-based thermally conductive composites.

## Graphical abstract

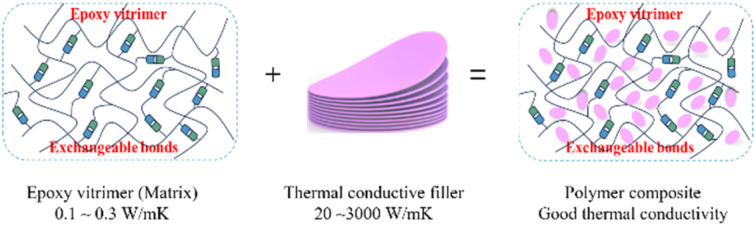

## 1. Introduction

Driven by the rapid development of the electronic information industry, electronic components are increasingly trending toward miniaturization, high integration, and high power. This progression has rendered heat dissipation a critical issue and technical barrier, impeding the further miniaturization and integration of microelectronic devices and electrical insulation equipment.^1,2^ Effective thermal management is a prerequisite for the further development of modern electronic devices.^3,4^ Currently, there is a high demand for thermal management materials (TMMs) in applications such as chips, light-emitting diodes (LEDs), 5G communication, and insulated gate bipolar transistors. The performance requirements for TMMs vary depending on specific application scenarios,^5–8^ with achieving the required TC and minimizing thermal resistance being decisive factors for efficient heat transfer.^9^ Polymer-based thermally conductive materials are a key component of the thermal management system for modern electronic devices. In recent years, researchers have conducted extensive studies on the design and preparation of high TC polymer-based composites, focusing on synthesizing polymer matrices with inherent high TC^10–16^ or incorporating inorganic thermally conductive fillers.^17–22^

Beyond high TC, TMMs also need to exhibit excellent comprehensive performance to meet application requirements. For instance, chip thermal interface materials (TIMs) require exceptional flexibility, elasticity, and interfacial adhesion to ensure effective gap filling.^23–25^ In certain specialized fields like optical devices, micromotors, and hard drives, TIMs need to exhibit sufficiently stability to prevent contamination by strictly limiting small molecular substances release during service.^26,27^ Consequently, silicone-sensitive applications necessitate silicone-free thermal pads synthesized from non-silicone polymers, which eliminate volatile silicone molecule and silicone oil precipitation issues.^28–30^ Epoxy resin (ER) can meet these stability demands through its adhesive strength, chemical resistance, and thermal stability, establishing it as a primary electronic packaging material.

Polymer-based TMMs face additional recycling challenges. Current TMMs including thermally conductive potting adhesives, thermally conductive phase change materials (PCMs), thermally conductive pads, and thermally conductive gels typically comprise permanently crosslinked thermosets.^31–33^ Their intrinsic irreversibility severely limits recyclability, necessitating disposal after use and causing environmental pollution.^34,35^ Moreover, high inorganic filler content in TMMs leads to resource wastage when discarded with the polymer matrix.^36–38^ Although widely used for thermal management bonding and potting,^39,40^ traditional ERs remain constrained by poor intrinsic TC (∼0.2 W m^−1^ K^−1^) and non-recyclability.^41^

The development of reprocessable “vitrimer” by Leibler^42–45^ addresses recyclability limitations. Epoxy vitrimers (EVs) behave like conventional ERs below their glass transition temperature (*T*_g_) but exhibit thermoplastic-like reprocessability above their topology-freezing transition temperature (*T*_v_), enabling reshaping and recycling.^46–48^ Vitrimers offer particular advantages in composites through their unique recyclability, absent in conventional systems,^49–52^ positioning them as sustainable matrix alternatives. The strategies for enhancing thermal conductivity of epoxy vitrimer-based composites are mainly divided into two categories: (i) the extrinsic approach by incorporating highly thermally conductive fillers, and (ii) the intrinsic approach by designing the molecular structure of the epoxy vitrimer matrix itself to achieve higher intrinsic thermal conductivity, for instance, by introducing liquid crystalline (LC) mesogens.^53^ While the former has been extensively studied, the latter represents a fundamental and promising strategy to create a high-performance matrix before compounding, potentially leading to synergistic effects when combined with fillers. Combining highly crosslinked EVs with oriented high-TC fillers (*e.g.*, boron nitride (BN), graphene, carbon nanotubes (CNTs)) produces recyclable thermally conductive composites (TCCs).^54,55^ Advancements in EV chemistry enhance composite recyclability, while optimized phonon transmission pathways improve matrix and composite TC.^56^ However, the vast majority of these reprocessing and self-healing functionalities require external heating to activate the dynamic bond exchange, which limits their application in scenarios where heating is impractical or energy-consuming. Therefore, developing epoxy vitrimer composites capable of room-temperature dynamic exchange while maintaining high thermal conductivity represents a highly desirable yet challenging frontier for next-generation smart thermal management materials.

This review surveys recent advances in EVTCCs. We first classify EVTCCs research by CAN type, summarize key characteristics (high TC, reshaping capability, recyclability), and outline future directions. These insights aim to guide the design of EVTCCs combining excellent thermal management with dynamic functionality, advancing sustainable thermal solutions.

## 2. Classification and synthesis strategies of EVTCCs

**Fig. 1:**
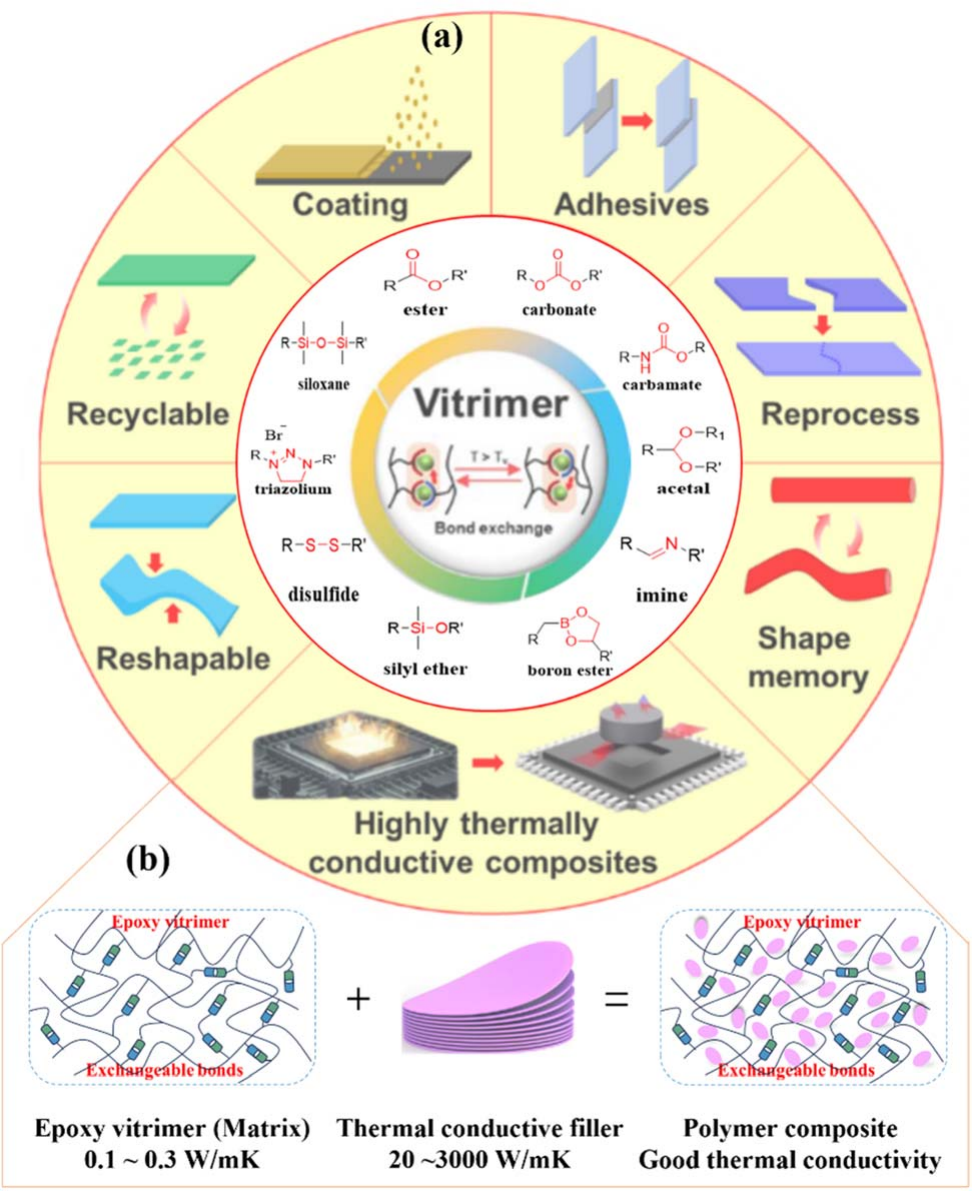
(a) Diagrammatic representation of dynamic covalent linkages employed in vitrimers and their applications; (b) EVTCCs are produced by integrating EV and TC fillers. Adapted with permission from ref. 58. Copyright 2024, MDPI.

Vitrimers exhibit bond-exchange capability through reversible reactions, enabling bond dissociation and reformation under external stimuli while conserving the total bond count. This unique behavior originates from their CANs formed during the preparation of vitrimers,^57,58^ encompassing ester,^59,60^ carbonate,^61^ carbamate,^62–64^ acetal,^65,66^ imine,^67,68^ boron ester,^69–72^ disulfide,^73^ triazolium,^74^ silyl ether,^75^ siloxane,^76^ and Diels–Alder diene addition reactions^77–79^ (Fig. 1a). Vitrimer multifunctionality combines stability with processability, enabling sustainable applications in reprocessable^80,81^ and recyclable polymers.^82,83^ They also facilitate adjustable and repairable coatings,^84^ adhesives,^85,86^ and reshapable materials.^87,88^ Their shape-memory properties show utility in 3D printing.^89–91^ Crucially, vitrimers play a important role in the field of composites,^49–52,92^ where their recyclability (a key advantage over conventional composites) enables diverse functional applications. This positions them as sustainable alternatives for TCCs (Fig. 1b). This review systematically classifies EVTCCs according to the dynamic CAN types within the EV matrix, with a focus on those that have been effectively utilized in TCCs. The discussion of synthesis strategies and bond-exchange mechanisms is intentionally centered on bond types that are most relevant to EV matrix and thermally conductive composite applications, reflecting the current state of the literature in this specific domain.

### 2.1 EVTCCs based on transesterification

**Fig. 2:**
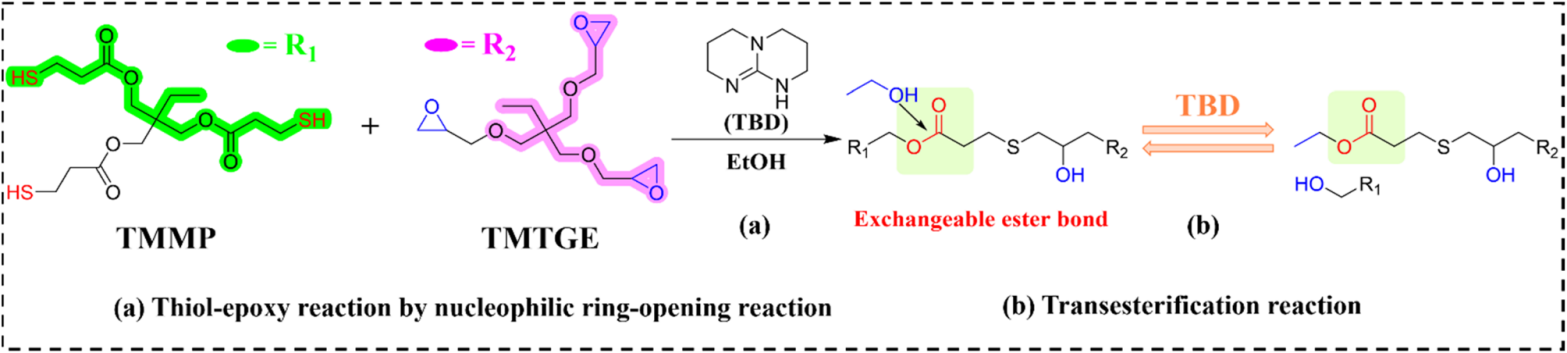
(a) Synthesis and (b) transesterification reactions in EVTCCs-1.

Transesterification-based vitrimers constitute the pioneering and most extensively studied category of vitrimer materials. Their dynamic behavior originates from catalyst-accelerated exchange between ester and alcohol functionalities, offering advantages of monomer accessibility and synthetic simplicity. However, significant limitations persist, including mandatory catalyst incorporation and high-temperature operation (typically >150 °C), restricting practical applications. These constraints notwithstanding, EVTCCs based on transesterification dominates current research. Yang *et al.*^93^ pioneered a thiol-epoxy vitrimer system using trimethylolpropane triglycidyl ether (TMTGE) and trimethylolpropane tris(3-mercaptopropionate) (TMMP) catalyzed by 1,5,7-triazabicyclo[4.4.0]dec-5-ene (TBD). Through nucleophilic ring-opening polymerization, *in situ* integration of micro-boron nitride (mBN), and hot-pressing, they fabricated self-healing recyclable composites (designated as EVTCCs-1; synthetic and transesterification reaction are shown in Fig. 2). At 60 wt% mBN loading, optimal TC (*λ* = 1.058 W m^−1^ K^−1^) was achieved, and 85% tensile strength maintained after self-healing, 70% tensile strength maintained following recycling and molding, and good thermal stability (*T*_HRI_ = 149.9 °C). Fortunately, the self-healing efficiency and effectiveness of the mBN/thiol-epoxy elastomer composites were seldom changed with the increasing addition of mBN fillers. One hand, the introduction of mBN fillers would impede the contact between the functional groups of transesterification reaction, against the efficiency and effectiveness of the self-healing. On the other hand, the improvement in *λ* value was beneficial to the promotion of self-healing systems relying on thermal response, to increase the self-healing efficiency and ensure the self-healing effect.

**Fig. 3:**
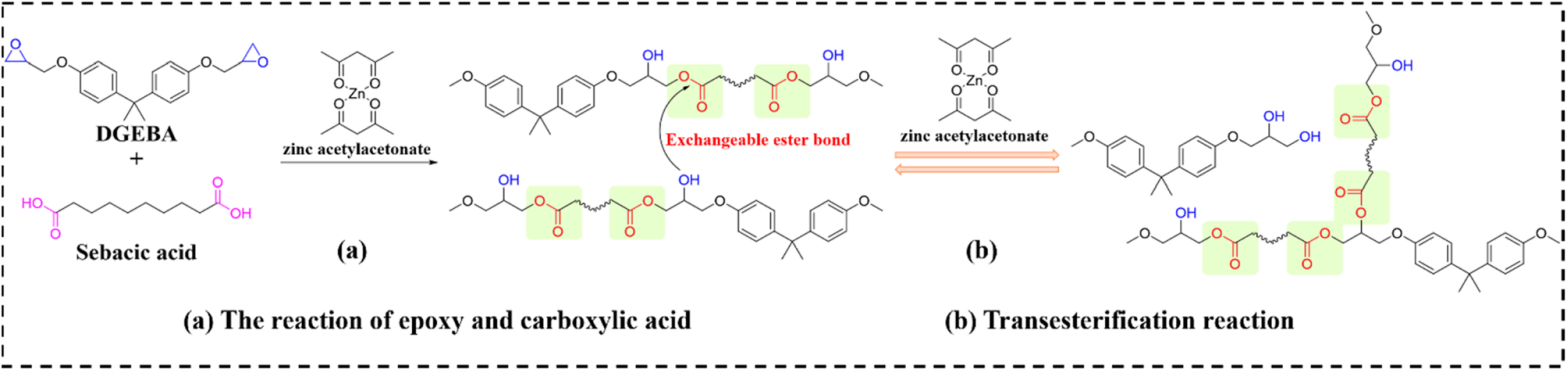
(a) Synthesis and (b) transesterification reactions in EVTCCs-2.

Carbon nanotubes (CNTs) and graphene suffer from poor dispersibility in polymer matrices due to strong van der Waals interactions and high aspect ratios. Poly(dopamine) (PDA) functionalization effectively enhances nanomaterial-polymer interfacial compatibility while preserving photothermal efficiency. Feng *et al.*^94^ developed EV-based carbon nanotube composites (designated as EVTCCs-2) *via* compression molding, using diglycidyl ether of bisphenol A (DGEBA) and sebacic acid (SA) catalyzed by zinc acetylacetonate. These composites demonstrate exceptional TC, mechanical strength, and stress relaxation behavior. Fig. 3 delineates the EV synthesis pathway and dynamic transesterification mechanism in EVTCCs-2. Critically, this work systematically interrogated how PDA-grafted multiwalled CNTs (MWCNTs@PDA) modulate composite morphology, TC, stress relaxation, and mechanical performance.

**Fig. 4:**
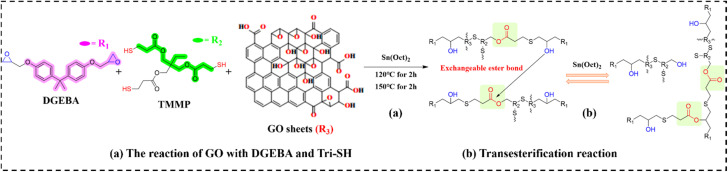
(a) Synthesis and (b) transesterification reactions in EVTCCs-3.

Graphene oxide (GO) demonstrates enhanced compatibility with epoxy vitrimers (EVs) due to its oxygen-functionalized surface (*e.g.*, carbonyl, carboxyl, hydroxyl, epoxide groups), while its cost-effectiveness distinguishes it among graphene-based materials. Vashchuk *et al.*^95^ fabricated novel EV nanocomposites (designated as EVTCCs-3) *via* thiol-epoxy click chemistry, utilizing DGEBA and TMMP with zinc acetate catalysis, incorporating ≤1.0 wt% GO. The trifunctional thiol cross-linker provides critical ester moieties for direct participation in dynamic transesterification (Fig. 4). Enhanced thiol reactivity (RSH *vs.* ROH) arises from the lower S–H bond enthalpy (*vs.* O–H), facilitating proton donation. TC quantification *via* gas-microphone photoacoustic measurements revealed a near two-fold enhancement at 1.0 wt% GO loading relative to the unfilled polymer matrix.

**Fig. 5:**
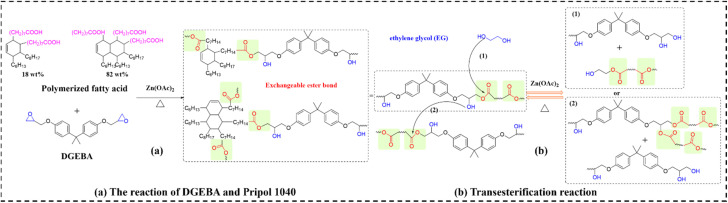
(a) Synthesis and (b) transesterification reactions in EVTCCs-4.

Polymer-based TCCs commonly employ graphene nanosheets, CNTs, silicon carbide, aluminum nitride, and hexagonal boron nitride (hBN) as fillers. Among these, CNTs (thermal conductivity: ∼3000 W m^−1^ K^−1^) and hBN (∼600 W m^−1^ K^−1^) dominate applications, though each presents distinct limitations: CNTs' high electrical conductivity precludes use in electrically insulating applications, while hBN's insulating character comes at the cost of lower TC. Conventional hBN composites require excessive filler loadings to achieve practical thermal performance, highlighting the critical need for advanced architectures that simultaneously optimize thermal transport and electrical insulation at minimal filler content. Zhang *et al.*^96^ addressed this challenge by developing EV composites with spatially segregated MWCNT and hBN networks *via* a two-step fabrication process. These composites (designated as EVTCCs-4) were synthesized from DGEBA and polymerized fatty acid using zinc acetate catalysis (Fig. 5). The key innovation lies in exploiting ethylene glycol (EG)-mediated alcoholysis and re-esterification at hBN interfaces, which accelerates dynamic exchange kinetics and enhances EV/MWCNT interfacial adhesion within the segregated hBN network. This architectural control yielded composites with 1 wt% MWCNTs and 8 wt% hBN that achieved TC of 0.83 W m^−1^ K^−1^ alongside electrical resistivity of 1.92 × 10^11^ Ω cm, demonstrating effective decoupling of thermal and electrical transport properties.

**Fig. 6:**
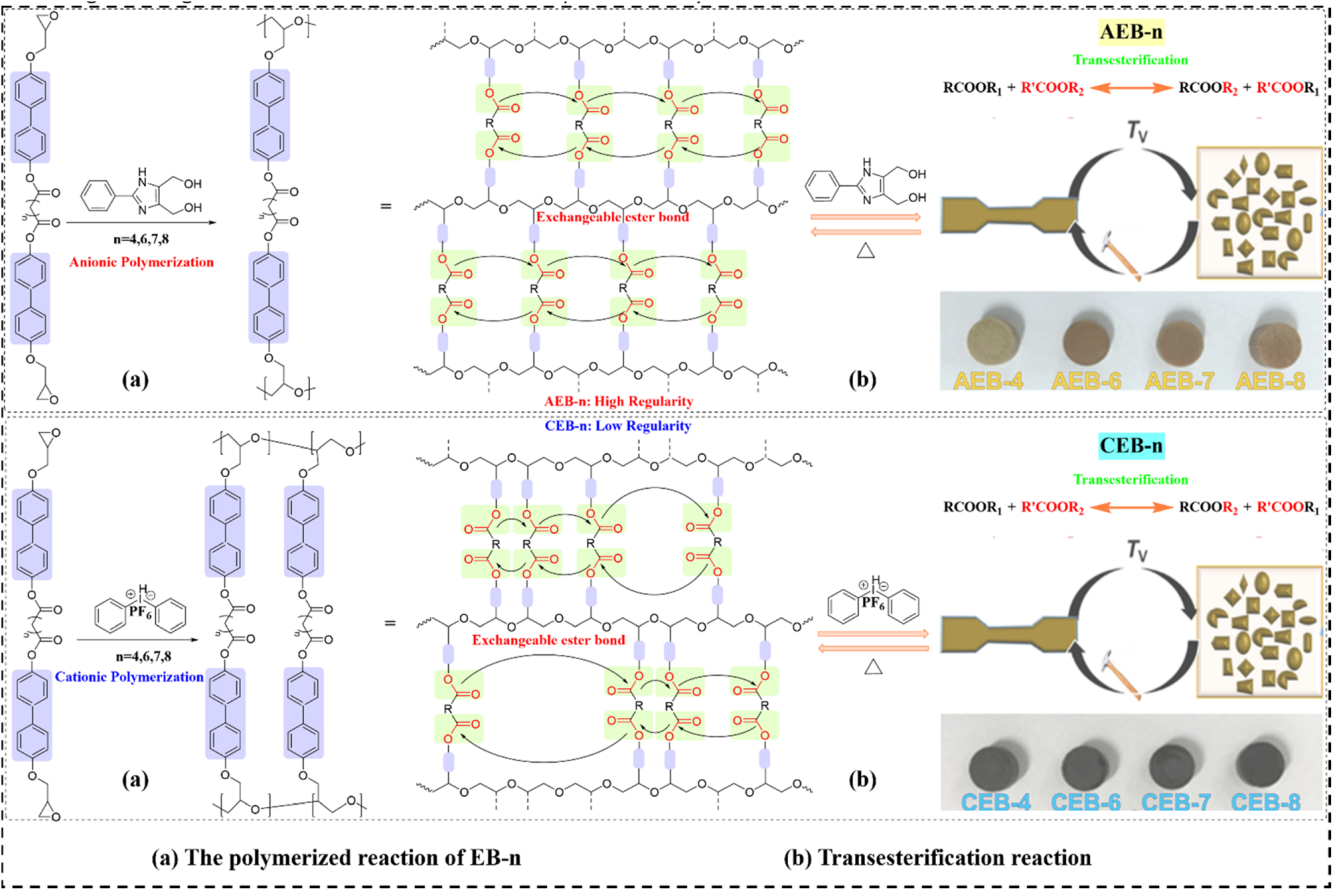
(a) Polymerization and (b) transesterification reactions in EVTCCs-5.

Conventional vitrimers, despite their attractive properties, typically require toxic external catalysts for transesterification-based reprocessing, causing material degradation and corrosion concerns. While thermally conductive fillers enhance the TC of polymer matrix, excessive loading creates interfacial thermal resistance (ITR) and compromises mechanical properties. Achieving high TC without sacrificing mechanical performance necessitates minimizing filler content while enhancing the matrix's intrinsic thermal transport. Trinh *et al.*^97^ developed catalyst-free liquid crystalline epoxy vitrimers (LCEVs) through rapid thermal polymerization of biphenyl mesogenic monomers (EB-*n*). Both anionic and cationic initiation systems enabled complete cure within minutes, with the polymerization and dynamic transesterification pathways illustrated in Fig. 6. Critically, the polymerization methodology dictated structural organization and resulting physical properties. These materials (for convenience in discussion, the mesogenic unit is treated similarly to a filler, and the synthesized LCEVs are designated as EVTCCs-5) achieved TC of ∼0.67 W m^−1^ K^−1^, a three-fold enhancement over conventional ERs (∼0.2 W m^−1^ K^−1^), while maintaining superior mechanical properties. Moreover, EVTCCs-5 demonstrated facile reprocessability *via* simple thermocompression without external catalysts, addressing the fundamental limitations of traditional vitrimer systems.

**Fig. 7:**
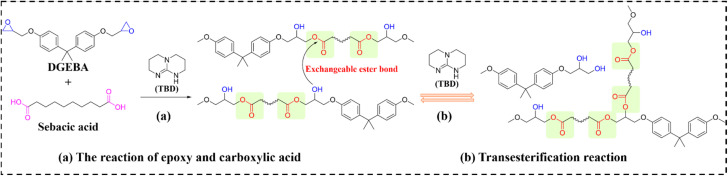
(a) Synthesis and (b) transesterification reactions in EVTCCs-6 and EVTCCs-7.

Enhancing the TC of ERs is essential for next-generation electronic-packaging materials. Microstructural engineering (including filler orientation within polymer matrices and constructing porous thermally conductive networks) provides an effective strategy to elevate TC in polymer-based composites. Cui *et al.*^98^ (Luo Fubin's group) synthesized EV/graphite flake (GR) TCCs (designated as EVTCCs-6) *via* DGEBA and SA catalyzed by TBD, which exhibit excellent self-healing and reprocessability. These composites exhibit exceptional self-healing and reprocessability, enabling film stacking through autonomous repair to construct bulk materials. Roll-induced alignment of GRs yielded a TC of 8.411 W m^−1^ K^−1^ at 50 wt% GR loading. Concurrently, Wang *et al.*^99^ (Luo Fubin's group) enhanced the flexibility of DGEBA/SA-derived vitrimers by integrating methoxy polyethylene glycol (MPEG) into the CAN. Increasing MPEG content improved ductility, achieving an elongation at break of 444.68%. BN incorporation produced EVTCCs-7, with 50 wt% BN elevating TC to 3.34 W m^−1^ K^−1^. Crucially, chemical decomposition of the CAN in EVTCCs-7 permits filler separation *via* filtration, enabling full matrix–filler recycling. Fig. 7 delineates the synthesis and dynamic transesterification in EVTCCs-6 and EVTCCs-7.

**Fig. 8:**
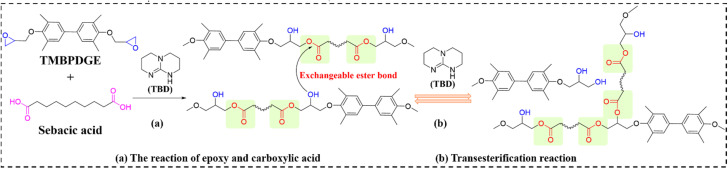
(a) Synthesis and (b) transesterification reactions in EVTCCs-8 and EVTCCs-9.

The escalating demand for TMMs has precipitated substantial depletion of petrochemical and inorganic mineral resources. Developing closed-loop recyclable composites with high TC is therefore critical. Leveraging the CAN concept, Luo Fubin *et al.*^100^ synthesized EV matrices using biphenyl ER and SA with TBD catalysis. Incorporation of BN and liquid metal (LM) fillers yielded recyclable biphenyl EV composites (designated as EVTCCs-8) exhibiting directionally controllable TC. High-power electronic architectures necessitate elastic TCCs for thermal management, whereas the inherent rigidity of conventional ERs intrinsically limits their utility as TMMs. Building upon EVTCCs-8, Luo's team^101^ introduced flexible polyethylene glycol (PEG) chains into the CAN, establishing a covalent–noncovalent interpenetrating network that imparted elasticity to the EV. Compared to traditional silicon-based TIMs, this elastic EV demonstrates superior reprocessability, self-healing, and no small molecule release. Remarkably, filler-incorporated derivatives (designated as EVTCCs-9) retained elasticity, reprocessability, and self-healing characteristics despite BN and LM loading. Synthetic routes and transesterification reactions for EVTCCs-8 and EVTCCs-9 are depicted in Fig. 8. Repeated calendering induced forced filler orientation, achieving reconstructed bulk composite TC values of 5.41 W m^−1^ K^−1^ (EVTCCs-8) and 3.66 W m^−1^ K^−1^ (EVTCCs-9) through thin-film stacking. Crucially, CAN-enabled separation of inorganic/organic constituents under mild conditions permits complete closed-loop recycling of both composite systems.

**Fig. 9:**
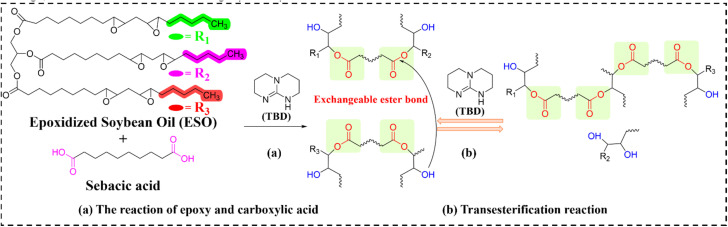
(a) Synthesis and (b) transesterification reactions in EVTCCs-10.

PCMs have garnered considerable interest as promising candidates for thermal management and energy storage applications. To develop a flexible, recyclable, and shape-stable thermally conductive PCM, Xu *et al.*^102^ from Luo Fubin's group utilized PEG as the phase-change component, which was confined within porous expanded graphite (EG) *via* vacuum adsorption. The dynamic crosslinked network formed by epoxy soybean oil (ESO) and SA conferred flexibility, prevented PEG leakage, and maintained structural integrity. The synthesized EV demonstrated thermoplastic-like reprocessability through transesterification reactions (as shown in Fig. 9). TC was further augmented by incorporating BN, yielding composites designated as EVTCCs-10. At a BN loading of 25 wt%, the TC reached 4.03 W m^−1^ K^−1^. The presence of dynamic covalent bonds enabled matrix degradation under mild conditions, facilitating the recovery of valuable thermally conductive fillers and enhancing material sustainability.

**Fig. 10:**
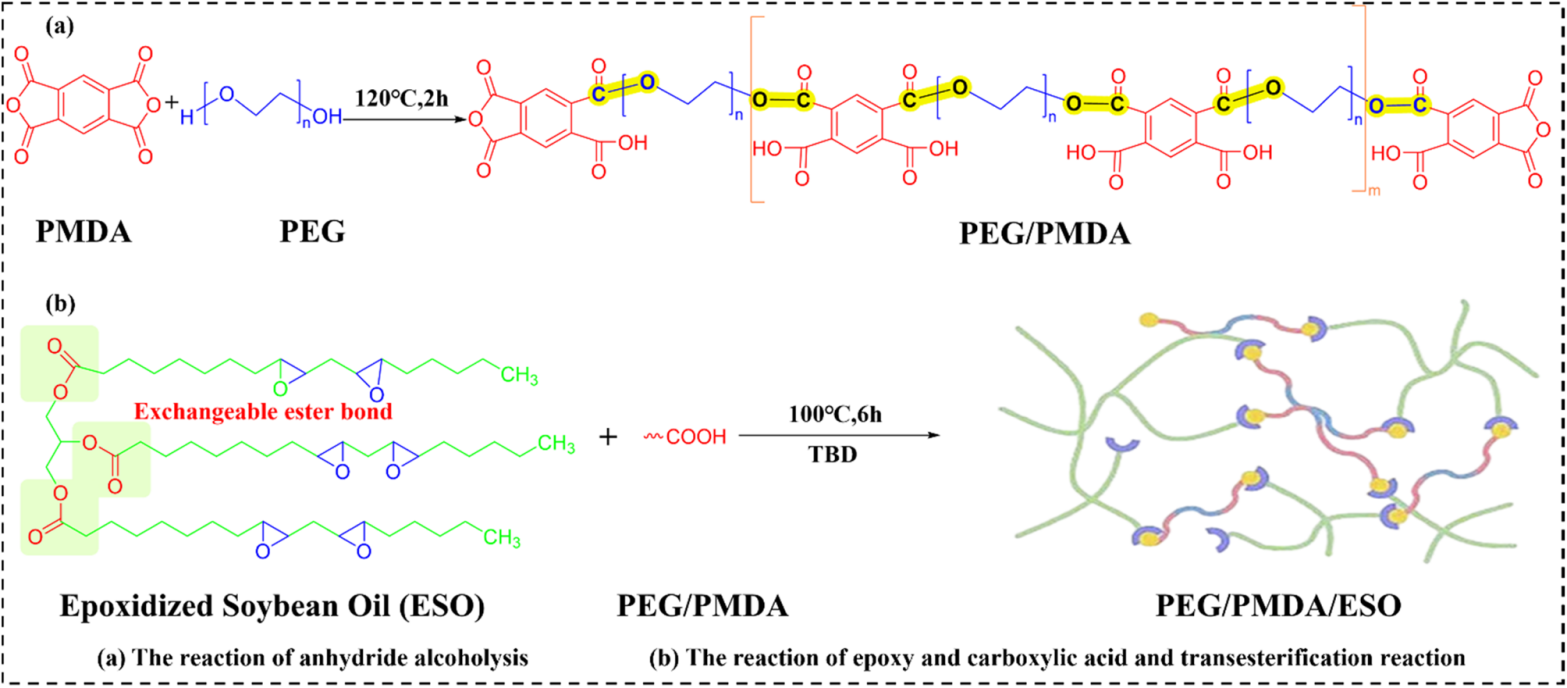
(a) The synthesis reaction of anhydride alcoholysis; (b) the reaction of epoxy and carboxylic acid and transesterification reaction in EVTCCs-11.

To develop environmentally friendly, recyclable, and shape-stable thermally conductive PCMs, Xu *et al.*^103^ also from Luo Fubin's group synthesized PEG-functionalized pyromellitic dianhydride (PEG/PMDA), a precursor containing phase change segments and reactive carboxyl groups. Subsequent co-curing of PEG/PMDA with ESO, catalyzed by TBD, yielded an EV featuring dynamic transesterification bonds (as shown in Fig. 10). This CAN simultaneously prevented PEG leakage and maintained structural integrity. BN incorporation significantly enhanced the TC of composites (designated as EVTCCs-11) reaching to 2.23 W m^−1^ K^−1^ at 40 wt% BN loading. Crucially, the dynamic bonds facilitated mild-condition matrix degradation, enabling closed-loop recovery of both conductive fillers and organic components.

### 2.2 EVTCCs based on disulfide exchange

**Fig. 11:**
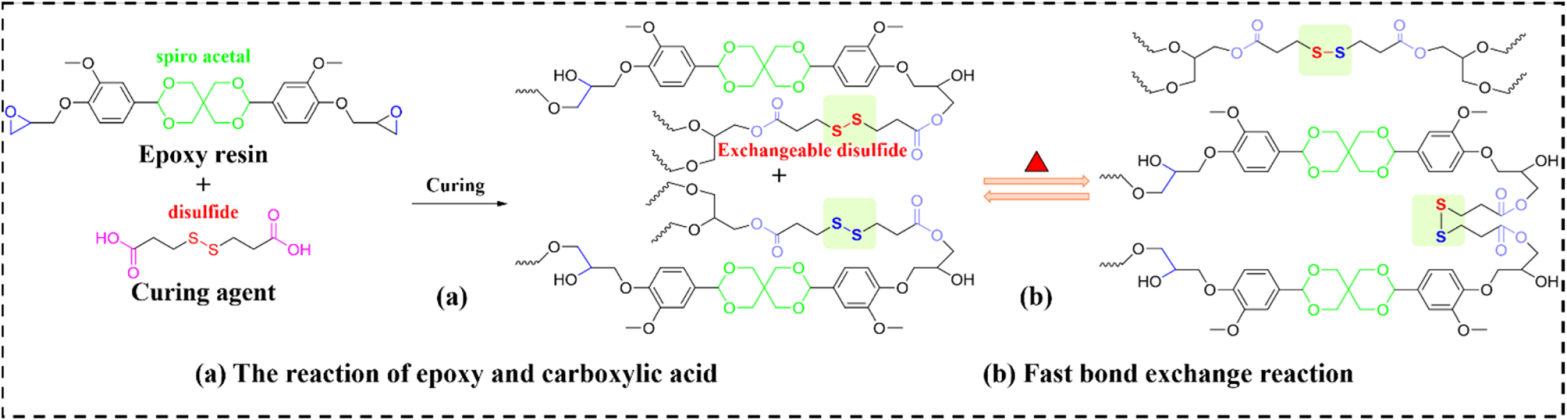
(a) Synthesis and (b) fast bond exchange reactions in EVTCCs-12.

Disulfide bonds represent a widely studied dynamic covalent bonds in self-healing polymers, owing to rapid ambient-temperature exchange kinetics and stimuli-responsive behavior (*e.g.*, heat, UV light). Liu *et al.*^104^ developed fully recyclable TIMs using a disulfide-functionalized EV and BN nanosheets (designated as EVTCCs-12). Synthesis and bond-exchange mechanisms are illustrated in Fig. 11. Hot pressing leveraged disulfide-enabled fluidity to achieve directional alignment of rigid BN fillers. This oriented, interconnected BN network established phonon transport highways, yielding anisotropic TC. At 40 wt% BN, TC reached to 3.85 W m^−1^ K^−1^. Which is 30-fold enhancement over pristine ER and 4.3-fold increase *versus* non-aligned composites. The material reduced core temperatures in electronics by 20 °C *versus* commercial silicones, attributable to its superior TC and interfacial conformability. Multilevel degradation pathways enabled efficient chemical recycling under mild conditions, with 96.2% BN and 73.6–82.4% organic feedstock recovery.

**Fig. 12:**
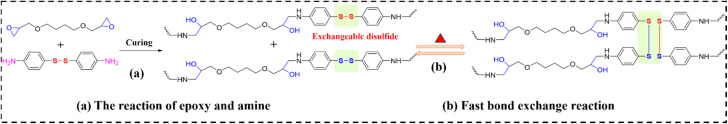
(a) Synthesis and (b) fast bond exchange reactions in EVTCCs-13.

EV-based composites with aligned fillers demonstrate significant potential for thermal management in high-power electronics, lowering operating temperatures while enhancing device stability and longevity. Wu *et al.*^105^ comprehensively characterized glass fibers (GFs) as fillers in EV matrices, evaluating their impact on mechanical properties, thermal transport, and disulfide exchange kinetics. The composites (designated as EVTCCs-13) were synthesized *via* a catalyst-free, solvent-free process (as shown in Fig. 12). Incorporation of 10 wt% GFs increased tensile strength by 4.37% *versus* neat EV, though higher loadings reduced toughness and delayed stress relaxation. Specifically, GFs impeded network dynamics, elevating the characteristic relaxation time at 160 °C from 15.35 s (pristine vitrimer) to 108.85 s (40 wt% GF composite). This filler alignment established directional phonon pathways, increasing thermal diffusivity by 110.17% at 40 wt% loading. Moreover, selective matrix degradation in dilute 1,2-dithiothreitol (DTT) enabled efficient GF recovery (>99% yield) without requiring surface functionalization for reuse.

**Fig. 13:**
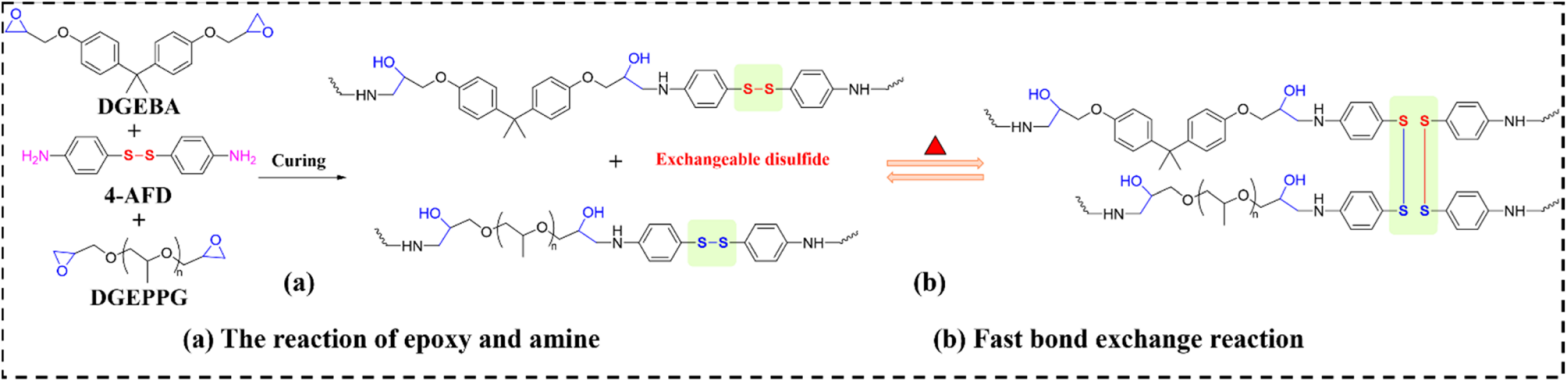
(a) Synthesis and (b) fast bond exchange reactions in EVTCCs-14.

Conventional mold casting techniques for EV-based composites constrain the geometric versatility required for intricate components. Additive manufacturing (AM) circumvents these limitations by enabling on-demand fabrication. Moreover, AM of thermally responsive shape memory polymers (SMPs) facilitates vertical integration across length scales, allowing the engineering of structural metamaterials that unify shape-memory behavior with bespoke mechanical and functional properties. In a seminal approach, Hong *et al.*^106^ synthesized a disulfide-functionalized EV through the reaction of DGEBA and poly(propylene glycol) diglycidyl ether (DGEPPG) using 4-aminophenyl disulfide (4-AFD) as the curing agent. This system exhibits pronounced shape-memory effects and reprocessability, attributable to dynamic disulfide bond exchange (as shown in Fig. 13). Feedstock inks comprising reduced graphene oxide (rGO) and hBN enabled direct ink writing (DIW) of EV-based composites (designated as EVTCCs-14). Incorporation of trace rGO (<0.5 wt%) yielded shear-thinning slurries with sufficient yield stress for ambient-temperature deposition of complex architectures. Introduction of hBN (≤22 vol%) and its shear-induced alignment during extrusion synergistically enhanced fracture resistance and induced anisotropic thermal transport. The resultant in-plane TC (3.0 W m^−1^ K^−1^) exceeded the matrix value by nearly an order of magnitude. This enhanced conductivity accelerates thermal actuation kinetics and enables the construction of hierarchical structures for precise heat-flow management when coupling with AM. Collectively, this work establishes a viable route toward additively manufactured thermoset shape-memory components, addressing persistent challenges in functional material design.

**Fig. 14:**
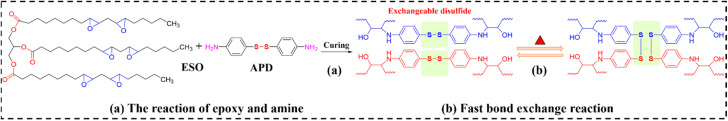
(a) Synthesis and (b) fast bond exchange reactions in EVTCCs-15.

Synthesizing ERs from biobased or hybrid feedstocks reduces fossil-resource dependence while mitigating environmental impacts. However, most conventional thermosetting ER TCCs remain non-recyclable post-service, causing substantial resource depletion and pollution. Sun *et al.*^107^ synthesized recyclable EVs using biobased ESO and 4-aminophenyl disulfide (APD) curing agents. Dynamic disulfide exchange enabled reprocessability while facilitating closed-loop recycling (as shown in Fig. 14). BN filler integration enhanced TC, with forced alignment during fabrication yielding directional composites (designated as EVTCCs-15). Optimized BN orientation achieved TC = 3.01 W m^−1^ K^−1^, 32.6% higher than randomly dispersed counterparts. Furthermore, disulfide-mediated matrix degradation under mild chemical conditions permitted complete filler separation *via* filtration, enabling direct BN reuse. This approach establishes a scalable, sustainable manufacturing pathway for bio-based TMMs.

### 2.3 EVTCCs based on imine bonds

**Fig. 15:**
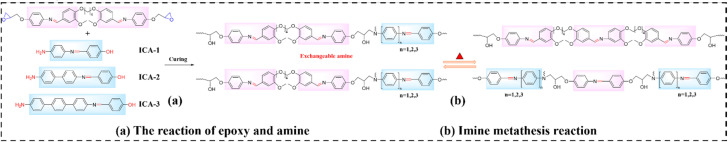
(a) Synthesis and (b) fast bond exchange reactions in EVTCCs-16.

Imine bonds have attracted significant research interest due to their synthetic accessibility, abundant precursor availability, acid–base responsive behavior, and mild dynamic exchange conditions. The vitrimeric bond exchange involving imines occurs *via* two distinct pathways: (i) nucleophilic addition between an imine group (C=N) and primary amine (NH_2_), generating new imine/amine pairs; (ii) imine metathesis between two imine bonds catalyzed by trace aliphatic/aromatic amines or metal species.^108^ Qin *et al.*^79^ synthesized a vanillin-derived liquid crystalline epoxy monomer (BEP) and three curing agents containing conjugated aromatic imine moieties (ICA-1, ICA-2, ICA-3). Subsequent reaction of BEP with each ICA produced LCEVs, whose synthetic routes and exchange mechanisms are illustrated in Fig. 15. These materials exhibited structure-dependent TC, with the conjugated length in ICAs critically influencing phonon transport. Relative to conventional ERs (0.23 W m^−1^ K^−1^), these LCEVs demonstrated enhanced intrinsic TC (0.28–0.38 W m^−1^ K^−1^) attributable to synergistic π–π stacking within the liquid crystalline phases of BEP and ICAs. Further TC augmentation was achieved through hBN incorporation (the resulting composites designated as EVTCCs-16). Crucially, the dynamic aromatic imine bonds enabled exceptional reprocessability *via* metathesis and quantitative matrix degradation in amine solutions.

### 2.4 EVTCCs based on multiple CAN

**Fig. 16:**
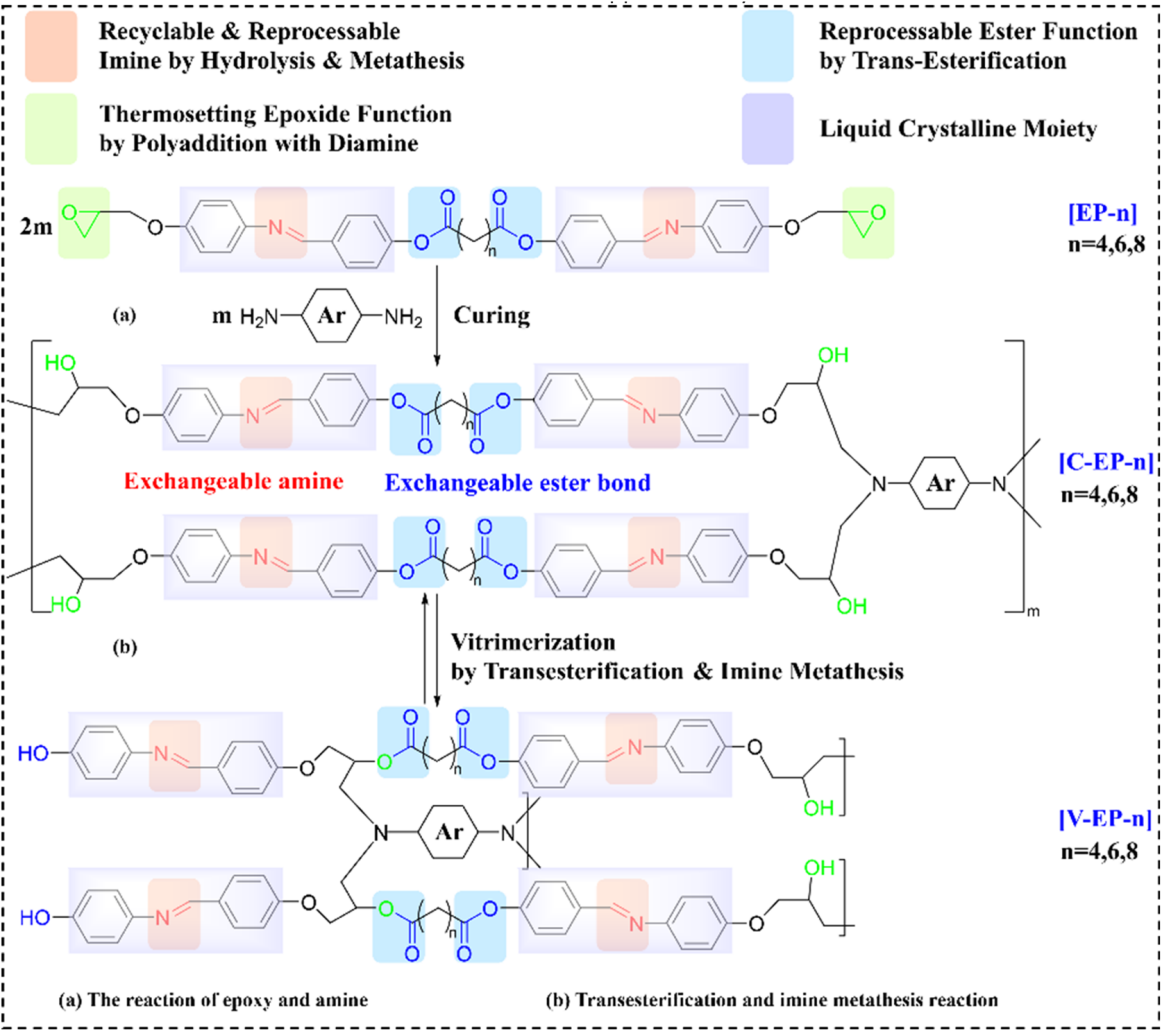
(a) Synthesis and (b) transesterification and imine metathesis reactions in EVTCCs-17.

The self-assembly of liquid crystal (LC) compounds is primarily driven by π–π interactions. Molecular design strategies for LCs typically involve aromatic-conjugated ester and imine functional groups. Trinh *et al.*^109^ synthesized bifunctional LCEVs with dual mesomorphic structures by tethering aromatic moieties to imine groups through ester linkages with variable aliphatic spacers. These compounds (designated as EVTCCs-17) were cured using conventional agents analogous to general-purpose ERs, yielding highly ordered structures with exceptional isotropic TC (up to 0.64 W m^−1^ K^−1^). Crucially, dual dynamic crosslinking sites within the vitrimer matrix enable catalyst-free reprocessing *via* ester exchange and imine metathesis at temperature above the *T*_g_, as depicted in Fig. 16. The cured products demonstrate facile reprocessability through imine metathesis and ester exchange. Under acidic or alkaline conditions, imine hydrolysis yields decomposable products suitable for recuring. Remarkably, recycled materials sustain multiple reprocessing or chemical decomposition cycles without performance degradation. Reprocessed variants retain the original material characteristics, while recycled products preserve the advantages of rigid thermosets without compromising functionality. During rehardening, dehydration reactions between residual hydroxyl groups elevate *T*_g_ by approximately 60 °C.

**Fig. 17:**
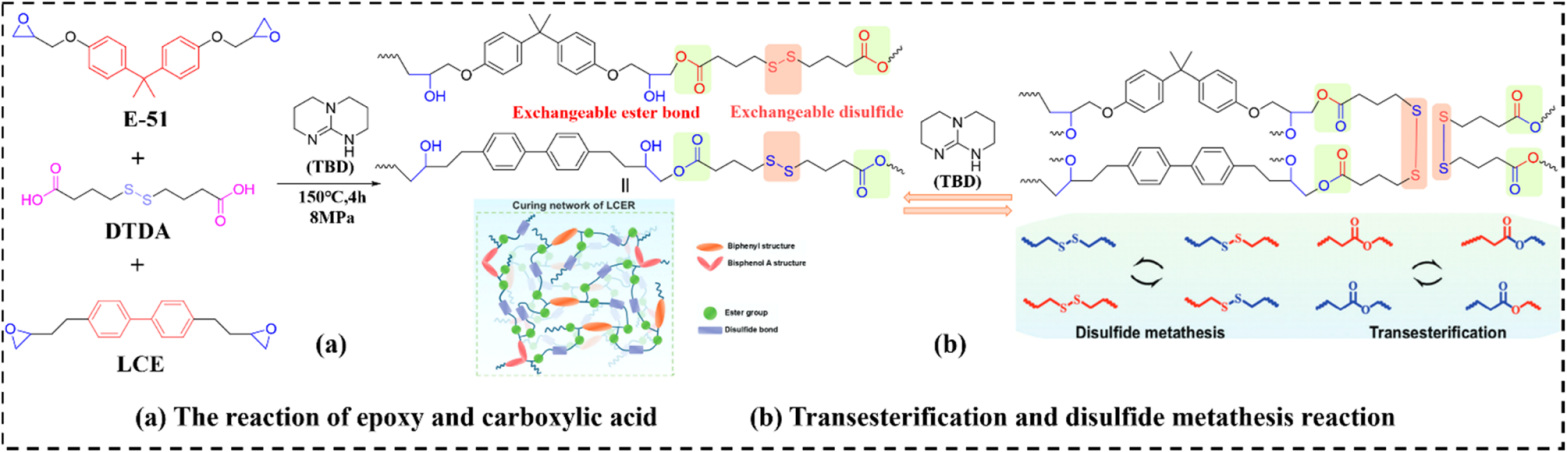
(a) Synthesis and (b) transesterification and disulfide metathesis reactions in EVTCCs-18.

LC units spontaneously assemble into oriented domains through π–π stacking interactions in molten or solution states. Incorporating such units to establish phonon conduction pathways represents an effective strategy for enhancing the intrinsic TC of eERs, while concurrently imparting superior mechanical properties and distinctive phase transition capabilities. Nevertheless, developing ERs that simultaneously exhibit high TC alongside excellent reprocessability and recyclability remains an outstanding challenge. Zhang *et al.*^110^ addressed this by integrating liquid crystal epoxy (LCE), dynamic ester bonds, and disulfide bonds into the DGEBA curing network, creating high TC flexible LCEVs (designated as EVTCCs-18; synthetic routes and bond exchange reactions shown in Fig. 17). Their study systematically investigated the influence of the LCE/DGEBA ratio on the intrinsic TC and mechanical properties of EVTCCs-18. EVTCCs-18 demonstrated exceptional reprocessability and shape memory characteristics. Notably, the inclusion of 0.5 wt% CNTs induced a pronounced photothermal effect. This enables NIR-mediated programming, autonomous self-healing, and interfacial welding capabilities, while further facilitating rapid degradation pathways and closed-loop recyclability.

## 3. Strategies and mechanisms for TC enhancement and functional synergy of EVTCCs

As summarized in Table S1, EV matrices incorporating dynamic CANs (including ester exchange, disulfide bonds, imine bonds, and multicomponent CANs), exhibit TC values spanning 0.16–0.61 W m^−1^ K^−1^. Integrating thermally conductive fillers or mesomorphic units markedly elevates composite TC to 0.34–8.41 W m^−1^ K^−1^. Key optimization strategies and their thermal conduction mechanisms are detailed below.

### 3.1 Synergistic filler strategy and mechanism

**Fig. 18:**
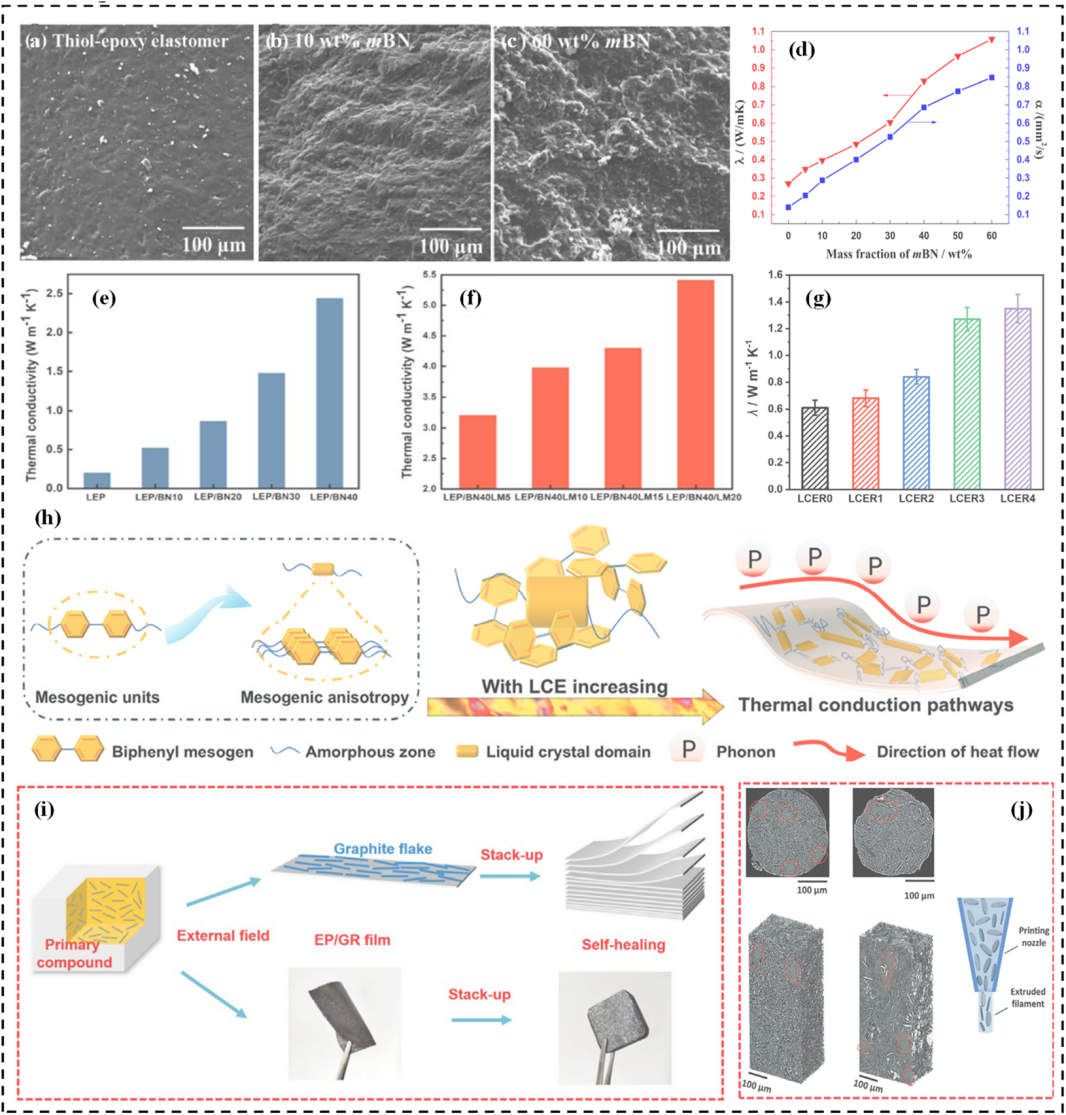
(a–c) SEM morphologies of the impact fractures for mBN/thiol-epoxy elastomer composites (500×). Adapted with permission from ref. 93. Copyright 2018, Elsevier. (d) *λ* and *α* values of the mBN/thiol-epoxy elastomer composites. Reprinted with permission from ref. 93. Copyright 2018, Elsevier. (e) Thermal conductivity of LEP filled with BN prepared by blending. (f) Thermal conductivity of LEP filled with BN and LM in the predominant direction. Reprinted with permission from ref. 100. Copyright 2023, Royal Society of Chemistry. (g) The *λ* values of LCER0-4. (h) Schematic of thermal conduction mechanism within LCER vitrimer. Reprinted with permission from ref. 110. Copyright 2024, Wiley-VCH Verlag. (i) Schematic diagram of the preparation of bulk vitrimer epoxy composites. Reprinted with permission from ref. 98. Copyright 2023, WILEY. (j) Illustration of the alignment of anisotropic platelets during extrusion through a thin nozzle through DIW. Reprinted with permission from ref. 106. Copyright 2024, Wiley-VCH Verlag.

High-loading inorganic fillers represent a primary strategy for TC enhancement. BN and its derivatives (*e.g.*, hBN and mBN) at 25–60 wt% yield composites with TC = 1.04–4.03 W m^−1^ K^−1^. At 50 wt% GR loading, a TC of 8.41 W m^−1^ K^−1^ (the highest reported value to date) is achieved. Lower addition of fillers was randomly distributed inner the matrix, and the fillers were commonly isolated and wrapped by matrix, hardly to form the thermally conductive channels. With the increasing addition of fillers, the fillers could contact with each other, to increase the formation probability of thermally conductive channels. And more thermally conductive channels & networks of the fillers–fillers through the matrix were formed with the further addition of thermally conductive fillers (Fig. 18a–d).

Conversely, dispersion limitations restrict low-dimensional fillers (*e.g.*, 1.0 wt% GO or 3.0 wt% MWCNTs@PDA) to modest TC improvements (0.34–0.47 W m^−1^ K^−1^). Synergistic filler strategy overcome these constraints: composites with 1 wt% MWCNTs and 8 wt% hBN reach 0.83 W m^−1^ K^−1^, while BN and LM hybrids optimize interfacial contact to achieve 3.66–5.41 W m^−1^ K^−1^ (Fig. 18e and f). A geometric synergy can be achieved by combining one-dimensional (1D) and two-dimensional (2D) fillers. A typical case is using a small amount of MWCNTs (1D) to bridge the gaps between larger hBN nanosheets (2D). The MWCNTs act as “conductive wires” that connect the isolated “conductive islands” of hBN, thereby forming a continuous 3D thermal network at a much lower total filler loading, which effectively enhances the thermal conductivity of the composite system. Furthermore, a more advanced strategy focuses on minimizing the interfacial thermal resistance, which is the greatest bottleneck in composites. This can be achieved by employing liquid metal (LM) as an interfacial mediator. The LM perfectly wets the surface of BN fillers, forming a continuous, metallic interfacial layer that “solders” the BN fillers together. This creates seamless phonon transport channels between fillers, drastically reducing the interfacial scattering and resulting in an exceptionally high thermal conductivity. This exemplifies how optimizing the filler–filler interface leads to a simultaneous enhancement of the entire composite's performance. The “synergistic filler strategy” is not merely an additive effect of the matrix and fillers, but rather a multiplicative synergistic effect achieved through ingenious filler combination and interface design.

### 3.2 Intrinsic enhancement strategy and mechanism

Incorporating mesomorphic units (*e.g.*, biphenyl, azobenzene) into the EV backbone intrinsically enhances matrix TC to 0.64–1.27 W m^−1^ K^−1^, substantially surpassing conventional matrices (∼0.20 W m^−1^ K^−1^) (Fig. 18g). The mechanism behind the dramatic enhancement is multifaceted: (i) formation of ordered phonon highways: the rigid, rod-like mesogens exhibit strong tendencies to self-assemble *via* π–π stacking interactions during the curing process. This leads to the formation of highly oriented, liquid crystalline domains within the crosslinked network. These domains act as low-resistance pathways for phonons, drastically increasing their mean free path compared to the disordered, tangled chains of conventional epoxy. (ii) Suppression of phonon scattering: the enhanced molecular order and the resulting more continuous structure minimize the disruptive interfaces between ordered and disordered regions that typically scatter phonons. This is a fundamental shift from a phonon-glass to a phonon-crystal-like structure within the polymer matrix. (iii) Enhanced intermolecular interactions: the strong π–π stacking not only drives the formation of ordered structures but also provides stronger intermolecular coupling in its own right. This facilitates the efficient hopping of phonons from one molecule to another, thereby enhancing the propagation speed of phonons (Fig. 18h).

### 3.3 Processing engineering strategy and mechanism

Hot pressing and hot curing constitute the predominant methods for preparing high-filler-loading EVTCCs. The TC of epoxy filled with ceramic fillers by blending is difficult to exceed 5 W m^−1^ K^−1^. Alternatively, film stacking and DIW enable ordered microstructure fabrication. For instance, the remolded composites through the rolling process for the preparation of thin film with 50 wt% GR achieve an in-plane TC of 8.411 W m^−1^ K^−1^. And DIW-processed composites with 22 vol% hBN attain an in-plane TC of 3.00 W m^−1^ K^−1^. The key mechanism is shear-induced alignment. For example: (i) the rolling process applies intense planar shear forces that act to align two-dimensional fillers like graphene (GR) parallel to the film plane. This creates highly ordered, laminated structures that provide a continuous, low-resistance pathway for phonon transport in the in-plane direction, resulting in very high TC values (Fig. 18i). (ii) Direct ink writing (DIW) utilizes the high shear rate within the printing nozzle to orient fillers (like hBN) along the extrusion direction (Fig. 18j). This process not only aligns fillers locally but also allows for the macroscopic design of thermal pathways, enabling the creation of composites with tailored anisotropic thermal properties. In both cases, the external mechanical energy input through processing overcomes the random distribution of fillers, actively constructing a well-oriented and interconnected network. This dramatically reduces phonon scattering at filler–filler interfaces and increases the effective phonon mean free path along the alignment direction.

### 3.4 Multi-scale synergistic design strategy and mechanism

The high performance of EVTCCs arises from a multi-scale synergistic design: (i) the dynamic covalent network serves as the foundational platform, enabling reprocessability and smart functionalities without compromising the material's structural integrity at service temperatures. (ii) Coexistence of function and high TC is achieved through a decoupled design: the filler network (and/or a mesogen-ordered matrix) provides efficient phonon transport, while the CAN matrix provides healability and adaptability. These two systems operate in parallel, minimizing interference. (iii) Recycling stability is ensured because the vitrimeric matrix can flow upon stimulation while the thermally conductive filler network remains intact or can be easily re-oriented, enabling closed-loop recycling of the composite itself. (iv) Active thermal management is made possible by leveraging vitrimer-specific properties like shape memory to improve interfacial contact and stress relaxation to ensure long-term durability against thermal cycling.

Ultimately, harmonizing high TC with vitrimer functionality is not a single-parameter optimization but requires a holistic approach that simultaneously considers filler topology (dimension/loading), interfacial engineering (*e.g.*, LM/BN synergy), matrix intrinsic conductivity (mesomorphic unit integration), and dynamic bond chemistry. This framework provides a deep mechanistic roadmap for the future design of intelligent thermal management materials.

### 3.5 Degradability and sustainable life-cycle management

Beyond reprocessability, an equally pivotal property of vitrimers is their controllable degradability. The dynamic covalent networks can be selectively cleaved under specific stimuli (*e.g.*, strong acids/bases, specific solvents, elevated temperatures), allowing the material to be broken down into its constituent parts at the end of its service life. This property is fundamental for achieving true sustainability and closed-loop recycling. For EVTCCs, this degradability offers a distinct advantage over conventional composites: the potential for efficient component recovery. Unlike traditional thermosets that yield hazardous waste upon harsh degradation, vitrimers can be degraded in a more controlled manner. (i) Reclamation of valuable fillers: the polymer matrix can be dissolved, allowing for the high-yield recovery and reuse of expensive conductive fillers (*e.g.*, boron nitride, graphene) without damaging their structure or performance. (ii) Recovery of monomers/oligomers: in some cases, the degradation products can be re-synthesized into new vitrimer matrices, closing the material loop completely.

However, a critical challenge lies in balancing degradability with long-term durability. The same labile bonds that enable end-of-life degradation could potentially lead to premature failure in hot and humid environments. Therefore, future research must focus on designing vitrimers with stimuli-responsive degradation, where the material is stable under service conditions but readily degrades only upon application of a specific, strong trigger. The integration of such intelligently designed degradable vitrimers with thermally conductive fillers represents a frontier in developing next-generation sustainable electronic materials.

## 4. Conclusion and outlook

### 4.1 Conclusion

In summary, this review comprehensively examines advances in EVTCCs. Through strategic design of CANs (*e.g.*, transesterification, disulfide exchange, imine bonds, and multiple CAN, *etc.*) combined with incorporation of thermally conductive fillers or liquid crystalline (LC) mesogens and optimized processing techniques, EVTCCs achieve synergistic enhancement of TC (0.34–8.41 W m^−1^ K^−1^) and dynamic functionality (self-healing, reprocessability, shape memory, self-welding, and physical/chemical recyclability, *etc.*). These materials represent next-generation candidates for electronic thermal management applications.

### 4.2 Outlook

Currently, research on epoxy vitrimer-based thermally conductive composites almost entirely relies on thermally activated dynamic exchange. The combination of highly thermally conductive fillers (such as BN, graphene) with epoxy vitrimer matrices that exhibit dynamic exchange at room temperature (such as systems based on disulfide bonds, imine bonds, and ionic bonds) remains unexplored. This is an emerging and critical cross-disciplinary area with significant challenges. The main challenges include: room-temperature dynamic chemistry (such as imine exchange, hydrolysis) may cause side reactions with the surface groups of the fillers, potentially disrupting the interface or the dynamic nature. How can a robust phonon transport interface be constructed in a dynamically active matrix at room temperature? Are traditional interfacial design strategies, such as silane coupling agents, still effective? Room-temperature dynamics typically require more “flexible” or mobile molecular chains, which may compromise the mechanical strength and modulus of the matrix, thereby affecting the overall mechanical properties and thermal conductivity stability of the composite. How can the long-term chemical stability and thermal durability of materials with dynamic properties at room temperature be ensured? Therefore, future work should focus on designing novel epoxy vitrimer matrices that utilize catalyzed exchange reactions (*e.g.*, transcarbamoylation with organocatalysts) or stimuli-responsive dynamic bonds (*e.g.*, disulfides, imines) that remain stable under service conditions but can be activated on demand at room temperature *via* specific triggers (*e.g.*, moisture, pH change, light). And the interface between fillers and these novel matrices requires completely new design principles, potentially employing dynamic covalent bonding at the interface itself to create adaptive yet thermally efficient interfaces. Overcoming these challenges will give rise to truly intelligent thermally conductive materials that can self-heal damage and self-adjust their shape to optimize thermal contact at room temperature, thereby significantly extending the lifespan of devices and enhancing the reliability and energy efficiency of thermal management systems. Photo-induced curing represents a promising strategy for manufacturing EVTCCs, particularly due to its ability to enable room-temperature processing and energy-efficient fabrication of complex parts. However, its application in high-filler-loading composites remains challenging due to severe light scattering, which hinders curing depth and uniformity. Future efforts should focus on innovative solutions such as photo-thermal dual-curing systems or the development of low-interference fillers to fully realize its potential for room-temperature processing of high-performance thermal management devices.

EVTCCs demonstrate exceptional promise for harmonizing performance, functionality, and sustainability. Future research should focus on exploring the synergy between the extrinsic approach by incorporating highly thermally conductive fillers and the intrinsic approach by designing the molecular structure of the epoxy vitrimer matrix itself to achieve higher intrinsic thermal conductivity. Designing novel liquid crystalline epoxy vitrimers that combine a highly ordered, thermally conductive network with dynamic covalent bonds represents a promising yet challenging frontier. Such matrices are expected to not only exhibit enhanced intrinsic thermal conductivity but also to form more efficient thermal pathways with incorporated fillers, ultimately leading to composites with superior overall performance, improved recyclability, and smart functionalities. The main challenges to be overcome include: dynamic exchange reactions (such as ester exchange) may disrupt the carefully constructed ordered liquid crystal structure; how to make the synthesis and curing process of liquid crystal monomers compatible with the preparation conditions of vitrimer. We believe that the highest performance epoxy vitrimer-based thermally conductive composites in the future will inevitably come from the synergy of intrinsic molecular design and extrinsic filler composites.

## Author contributions

Huimei Yao: investigation, data curation, writing – original draft; project administration; funding; supervision. Xuan Xu: investigation, data curation; Zipeng Chen, Kai Li, Tao Wang, Shenghua Pei, Jiaojiao Fu, Jiaxin Wang: writing – review and editing. All authors reviewed the manuscript.

## Conflicts of interest

The authors declare that they have no known competing financial interests.

## Supplementary Material

RA-015-D5RA05874K-s001

## Data Availability

No primary research results, software or code have been included and no new data were generated or analysed as part of this review. Supplementary information is available. See DOI: https://doi.org/10.1039/d5ra05874k.
