# Supplementary material for: A review of epoxy vitrimer-based thermally conductive composites
Source: RSC Adv. 2025 Oct 10;15(45):37908–25. doi: 10.1039/d5ra05874k (PMC12513471; doi:10.1039/d5ra05874k)
Supplement: RA-015-D5RA05874K-s001 [file RA-015-D5RA05874K-s001.pdf]

Supplementary Information (SI) for RSC Advances.
This journal is © The Royal Society of Chemistry 2025

A Review of Epoxy Vitrimer-based Thermally Conductive Composites

Supplementary Information

Table S1. The TC and vitrimer performances in EVTCCs

EV classification	Preparation method	Polymer matrix TC (W/m·K)	Filler contents	Composite TC (W/m·K)	Vitrimer performances	Ref.
Transesterification	Hot pressing method	0.27	mBN: 60 wt%	1.058	(1) Self-healing<br>(2) Physical recyclability	93
	Hot pressing method	0.16	MWCNTs@PDA: 3.0 wt%	0.34	(1) Stress relaxation behavior<br>(2) Shape memory<br>(3) Repairing properties<br>(4) Physical recyclability	94
	Hot curing method	~0.27	GO: 1.0 wt%	0.47	/	95
	Hot pressing method	0.23	MWCNTs: 1 wt%<br>hBN: 8 wt%	0.83	(1) Stress relaxation behavior<br>(2) Reprocessing performance	96
	Hot curing method	~0.20	biphenyl mesogen	0.68	(1) Stress relaxation behavior<br>(2) Reprocessing performance	97
	Remolding by self-healing	~0.20	Graphite (GR): 50 wt%	8.411	(1) Self-healing<br>(2) Reprocessing performance	98
	Hot curing method	0.23	BN: 50 wt%	3.34	(1) Self-repairing<br>(2) Physical/Chemical recyclability	99
	Film stacking method	~0.20	BN: 40 wt%<br>LM: 20 wt%	5.41	(1) Self-healing<br>(2) Physical/Chemical recyclability	100
	Film stacking method	~0.20	BN: 30 wt%<br>LM: 30 wt%	3.66	(1) Self-healing<br>(2) Physical/Chemical recyclability	102
	Hot curing method	~0.20	BN: 25 wt%	4.03	(1) Excellent interfacial adaptability<br>(2) Chemical recyclability	102
	Hot curing method	~0.20	BN: 40 wt%	2.23	(1) Shape memory<br>(2) Chemical recyclability	103
Disulfide	Hot pressing method	~0.20	BN: 40 wt%	3.85	(1) Excellent interfacial adaptability<br>(2) Chemical recyclability	104
	Hot curing method	/	GFs: 40 wt%	significantly increased	(1) Stress relaxation behavior<br>(2) Chemical recyclability	105
	DIW	0.37	hBN: 22 vol%	3.00	(1) Printability<br>(2) Reprocessability<br>(3) Shape memory	106
	Film stacking method	0.205	BN: 35 wt%	3.01	(1) Self-healing<br>(2) Physical/Chemical recyclability	107
Imine	Hot curing method	0.23	hBN: 40 wt%	1.04	(1) Reprocessability<br>(2) Chemical recyclability	79
Multiple CAN	Hot curing	~0.20	a twin mesogenic	~0.64	(1) Reprocessability	109

	method		structure		(2) Chemical recyclability	
	Hot pressing method	0.61	Biphenyl mesogens	1.27	(1) Reprocessability<br>(2) Shape memory<br>(3) Self-healing<br>(4) Self-welding<br>(5) Chemical recyclability	110
